# Mandatory Check for COPMI in Adult Mental Healthcare Services in the Netherlands—A Quantitative and Qualitative Evaluation

**DOI:** 10.3389/fpsyt.2022.807251

**Published:** 2022-03-18

**Authors:** Saskia Everts, Therese van Amelsvoort, Sophie Leijdesdorff

**Affiliations:** ^1^Division Child and Adolescent Mental Health, Mondriaan Mental Health Center, Maastricht, Netherlands; ^2^Department of Psychiatry and Neuropsychology, Maastricht University, Maastricht, Netherlands

**Keywords:** COPMI, children, parental mental illness, mental disorders, intervention, mandatory check, prevention

## Abstract

**Background:**

Children of parents with a mental disorder and/or addiction (COPMI) are at increased risk of developing a mental disorder. In spite of preventive interventions that can decrease the risk of problem development, COPMI are not automatically offered help. In 2013, a mandatory COPMI check was implemented in the Netherlands, requiring every mental health care professional to check whether their adult patients have children and to assess these children's safety and needs. Earlier research has shown that a gap between these regulations and the actual integration in clinical practice is not uncommon.

**Method:**

In the current study, we evaluated the implementation of the mandatory COPMI check in the Netherlands, using quantitative as well as qualitative data from a large mental healthcare organization in the Netherlands that offers both Child and Adolescent Mental Health and Adult Mental Healthcare.

**Results:**

Files from 14,469 patients were analyzed quantitatively and a sample of 150 files was further analyzed in depth. Findings were refined through 4 focus groups with adult mental healthcare professionals. It was found that while there are examples of the tool leading to interventions for COPMI, the tool is often not used, and when used tends to direct the focus away from COPMI needs and organizing help toward the more narrow and problematic focus on safety and reporting to child abuse authorities.

**Conclusion:**

The potential of the COPMI check is currently not fully realized. Strategies to improve its effectiveness in clinical practice are needed to improve access to interventions for COPMI.

## Introduction

It has been well documented that children of parents with a mental disorder and/or addiction (COPMI) are at considerable risk to develop mental disorders themselves (1–3). Accounting for this risk are both hereditary factors, as well as a potential inadequate developmental context (including child-abuse) that can arise when parents suffer from mental disorders or addiction. Moreover, congenital factors within the child and contextual factors in the family, influence each other as well (4).

Of course, not all children whose parents struggle with mental illness will develop mental problems themselves, nor will their development necessarily be problematic. There is quite some important literature on the resilience of children and on protective factors that can counter the risk of having a vulnerable parent (5, 6). Also, several Child and Adolescent Mental Health interventions exist to increase resilience of children and mitigate potentially negative developmental effects among COPMI (7, 8). These include programs directly targeting both children and parent(s) regarding their parenting tasks. Many of these interventions are found to be evidence-based (9–11) and/or are experienced by professionals and children or their parents as helpful (12, 13). Moreover, it has been established that early and preventive interventions can decrease the risk of problem development in COPMI with 40% (14, 15). One of the key questions is how these available interventions can be brought timely to those needing it. As COPMI are at greater risk, approaching them through their mentally ill caregivers (usually the parents) could provide an entry point for (early) detection and intervention. Therefore, organizations providing mental healthcare to parents can play an important role in the identification of those children at risk, enabling prevention and (early) intervention through specific programs for COPMI (16). This role has however not typically been taken up spontaneously by mental healthcare organizations or individual professionals and appears not easy to fulfill (17–20), partially due to different ways of working between child and adolescent mental health services (CAHMS) and adult mental health services (AMHS). Therefore professionals working with adults with mental disorders should be encouraged to play a role in enabling COPMI to access help. In a few countries, notably Norway and the Netherlands, as well as in the state of Victoria in Australia (21), a top-down approach has been chosen aimed at a routine identification of COPMI in adult mental health services: attention to COPMI has been required by law and tools are introduced that should be used routinely. The Norwegian COPMI project has been evaluated at different stages, showing that in the first 3 years after implementation, significantly more children were identified as being at risk, yet follow-up in terms of support for these children did not significantly increase (18). A follow up study found that after 5 years there was little increase in experience, attitude and knowledge or experience with family conversations among adult health care workers about COPMI (22). Similar limited results were found in Australia (21).

In the Netherlands, both the obligation to ask about the children of adult patients receiving mental health care (MHC) as well as an instrument to facilitate this mandatory check (*Kindcheck* or COPMI check), were introduced in 2013 (23). The present study (1) evaluates the implementation of this COPMI tool, (2) explores whether this has resulted in increased support for COPMI and (3) identifies potential strengths and barriers.

### COPMI Check

The Dutch COPMI check was originally developed, and is still presented as part of a nationally implemented protocol aimed at reducing the incidence of child abuse and domestic violence (24, 25). This so called Reporting Protocol offers a five-step decision tree, detailing the best course of action in case of suspected child abuse or domestic violence. The COPMI check is presented as part of the first step, which is to document the warning signals that support or contradict such a suspicion. The COPMI check focuses on the “parental warning signals” that may indicate risk for child abuse, which include the (mental) health issues of the parent. The tool is meant to be used by professionals working in adult health care.

It is of note that the COPMI check was introduced with a focus on child *abuse*, while the present study is concerned with the broader issue of *mental health needs* and well-being of children and adolescents at risk. Child abuse or safety can be seen as one extreme of a continuum, with general mental and developmental needs of children at the other end. Of course the distinction between safety and mental health needs is gradual and the two foci overlap. For example, the broader issue of emotional neglect is often included in the official definitions of child abuse violence^1^ (26). But although there is overlap, there is still a clear difference between the two ends of the continuum, with the COPMI check focused on the safety end. Despite this differing focus, it would seem reasonable to expect and hope that the COPMI check, as the only mandatory and widely implemented tool addressing COPMI, would contribute to an increased support for COPMI both regarding abuse and regarding broader mental health needs. An evaluation of the Dutch COPMI check has not been done from this perspective before, although reference to problems with its use are made in some Dutch studies (27–29). Thus, the present study evaluated the implementation, use and outcome of the COPMI check at Mondriaan Mental Health Center, a large mental healthcare organization in the South of the Netherlands, that incorporated the COPMI check in 2016 as mandatory tool in their standard intake assessment procedure.

## Materials and Methods

### Patient Files

#### Subjects

Data were collected prospectively for a period of 4 years; between December 2016 (start of the implementation) and January 2021. To include a group of patients with a reasonable chance of having the responsibility over underage children, patients outside the age range of 20–65 years were excluded. As a result, completed COPMI checks of 14.469 patients were retrieved from electronic patient files. This data set was anonymized. The study protocol and procedure were assessed as non-invasive and approved by the medical ethical committee of Maastricht University (protocol number: 2021-2784).

#### Measures

The COPMI check was operationalized at Mondriaan Mental Health Center as a brief tool, existing of one question with three answering possibilities, integrated in the standard intake formats. The quantitative data of this study are the answers to this COPMI check question.

COPMI check question: Has the COPMI check been carried out?

1. Yes, no risk present
2. Yes, risk present (if so: put relevant information in COPMI-check form and record interventions in treatment plan)
3. No, not carried out (with follow-up question: Why not?)

If none of the boxes was ticked, we consider this category 0, described as “No answer given”.

#### Procedure and Analysis

For each of the first two categories (responses 1 and 2), a random sample of 75 patient files was taken, using Excel, version 16.50 (Microsoft, Redmond, WA, USA). These patient files were further investigated to (1) extract in-depth information about how professionals came to their answers to the COPMI check question, and (2) whether they initiated further steps such as organizing or providing some form of help. For each file, a summary of the available information on the children, the home-situation, the COPMI check and related considerations was written. Following thematic analysis, these summaries were categorized into different themes relevant to understanding the decisions made by the professionals.

### Focus Groups

#### Setting

To help interpret and enrich the results from the analysis of patient files, four focus groups were conducted. We made use of the regular team meetings of AMHS professionals, which we joined for 30–90 min in order to conduct a focus group discussion regarding our research questions. Participants were contacted in advance with comprehensive study information. Withdrawal from participation of the study was possible at any stage in the process which was repeatedly stated by the researchers. Oral informed consent was obtained. Focus groups were recorded with an audio-recorder and later transcribed to text in their original language. Any relevant notes made during the focus group were included in the transcript as well. At the end of each focus group, the researchers did a “member check” (30) by summarizing the discussion and asking participants to either adjust, add to or approve this summary.

#### Participants

To gain insight into the full scope of barriers and facilitators, we joined the meetings of different AMHS teams thus using a form of purposeful sampling (31). The following meetings were joined and used for a focus group session:

1. A routine multidisciplinary meeting from the team working on Anxiety, Compulsion, and Trauma.
2. A routine multidisciplinary meeting from the team working with Attention Deficit Hyperactivity Disorder and Autism Spectrum Disorders.
3. A routine meeting to discuss possible crises, in which AMHS professionals working with Anxiety disorders, Psycho trauma, and Personality Disorders participated.
4. A quarterly meeting of AMHS professionals that function as “internal ambassadors” for the implementation of the Reporting Protocol Domestic Violence and Child Abuse. These internal ambassadors are assigned to stimulate the use of the COPMI check and broader protocol of the Reporting Protocol Domestic Violence and Child Abuse.

By joining routine meetings, we were able to reach more AMHS professionals, as well as a more random selection of them, than if we would have scheduled separate focus group meetings. The teams were heterogeneous regarding expertise, treatment options, and department within the organization. Most AMHS professionals worked directly as therapists with adult out-patients in the domains mentioned above. Some others worked on an internship basis and had few independent contacts with patients. In one of the team meetings a psychiatrist was present. The majority were women, reflecting the gendered division of labor in this sector. Inclusion and data analysis ran in parallel, providing a constant feedback-loop between both processes. To provide adequate depth on this topic, inclusion was continued until no new insights with respect to the main research questions emerged (32, 33).

#### Procedure and Analysis

Because of the COVID-19 pandemic, all focus groups were held remotely. Focus groups were conducted in Dutch. Researchers SE and SL led all focus groups, alternating a leading or observational role. As participants were direct colleagues and thereby familiar with each other, focus groups started with researchers introducing themselves and the study. Next, two questions were asked, both oral and written in the chat function of the online environment. We used a variant of the 1–2–4-All technique (34), that is participants were asked to take 2 min to formulate their individual answer to these questions, followed by a brief discussion of another 2 min of their answers with one other participant in break-out rooms. Remaining time was used for a group discussion followed by a short summary by the researchers and a possibility for remaining questions and remarks. Researchers used probing questions to reach more in-depth answers. Inductive thematic analysis was conducted (32, 33). Analysis started with an explorative phase of open coding, in which basic themes were defined, followed by merging these themes into more conceptual categories, and after the major topics were identified, codes were analyzed further identifying the most important themes.

## Results

### Use of the COPMI Check

**Figure 1:**
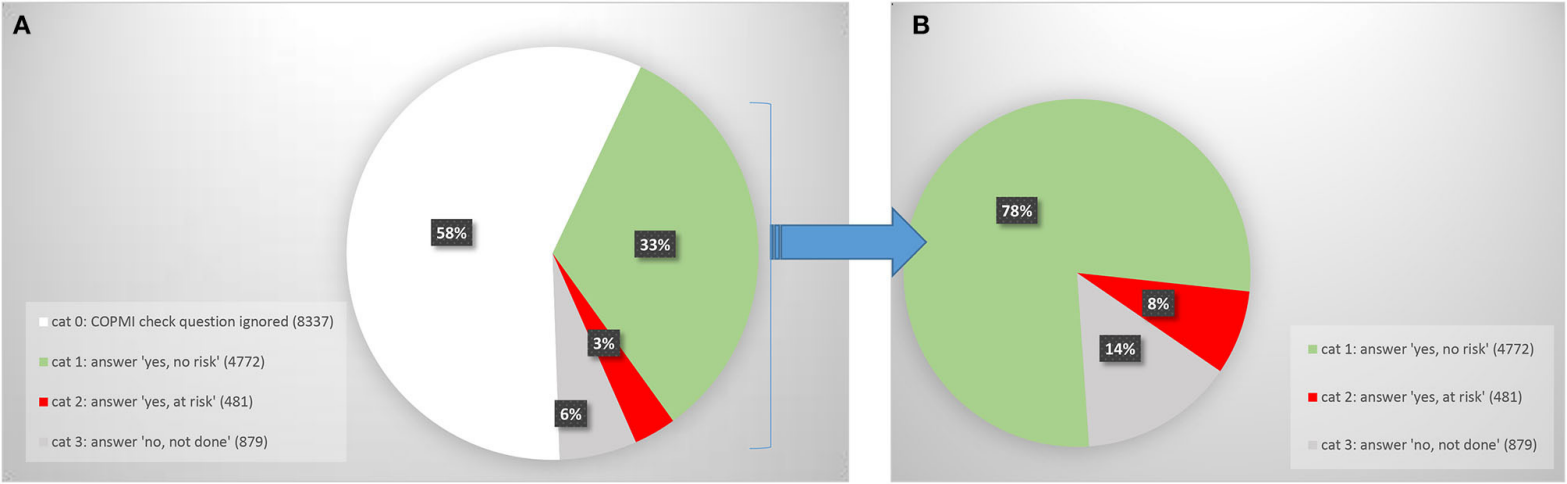
**(A)** Answer to COPMI check question: all patients (*N* = 14,469). **(B)** Answer to COPMI check question: patients for whom the question was answered (*N* = 6,132).

Figure 1A shows the way professionals dealt with the COPMI question for the total of 14,469 patients aged 20–65 that were treated by Mondriaan between December 2016 and January 2021.

For 58% of the 14,469 patients aged 20–65, the COPMI check instrument was not used at all, that is: none of the three answering categories to the COPMI check question were selected. We call this category 0.

Figure 1B quantifies how professionals responded to the COPMI check question when they did not ignore it. 78% of these patients was judged to be *not at risk* regarding their children (category 1), while 8% was judged to be *at risk* regarding their children (category 2). Category 3 (14%) exists of patients for whom the professional answered the COPMI question by saying they did *not do* the COPMI check.

It is important to note however that category 1 (judged not to be at risk regarding their children: 78%) includes many patients who simply do not have children. Thus, to interpret the figures, it is necessary to distinguish between patients who are and who are not caregivers for underage children.

### Caregivers vs. Non-caregivers

Inspection of the data revealed that when patients did *not* have children, the question “have you carried out the COPMI check” created confusion for the professionals. When their patient was not a caregiver, some professionals responded that they had done the COPMI check but “no risk” exists (category 1) because no children are present. Yet others responded that they have “not carried out” the COPMI check (category 3) because no children were present. This is a result of an apparent multi-interpretability of the COPMI check question. This inconsistency in interpretation makes it necessary to quantify the number of caregivers within each category.

**Figure 2:**
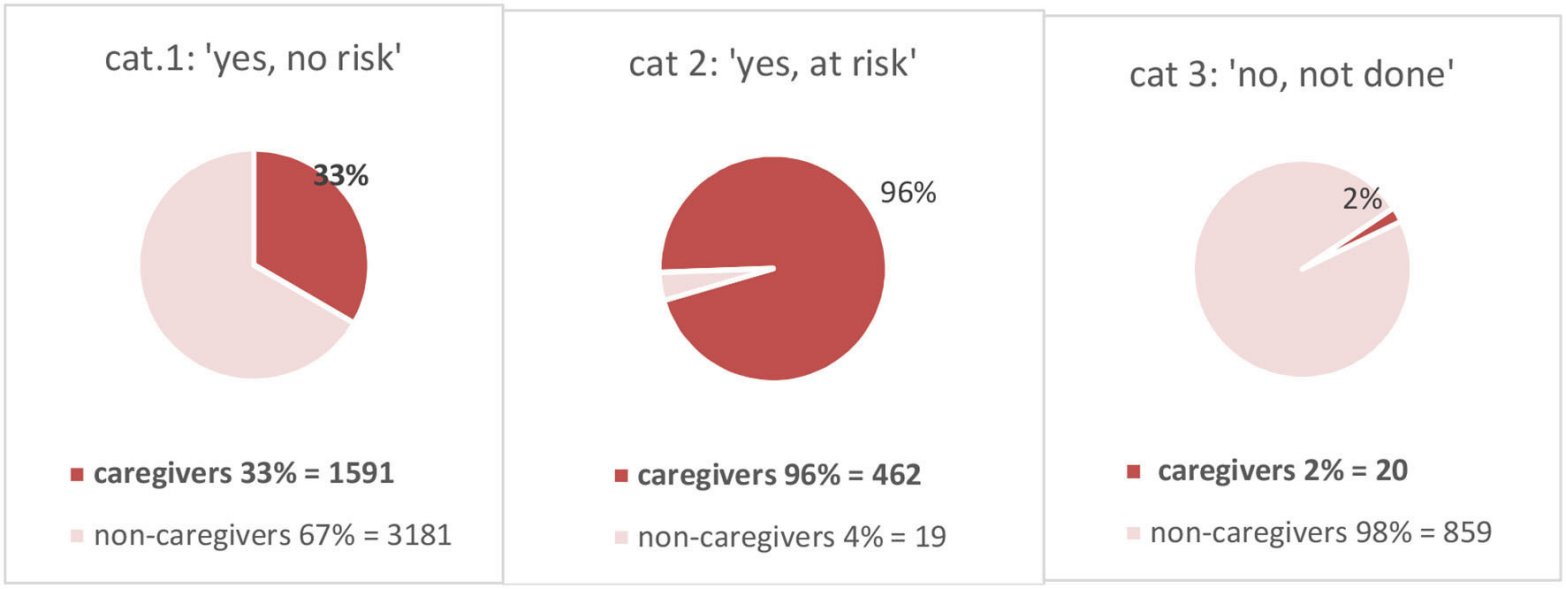
Parenting status for each category.

We checked parenting status in the representative sample of *N* = 75 taken from all patients in categories 1 and 2, the results of which were extrapolated to all patients in those two categories. In addition, we did a visual check of all patients in category 3. Parenting status is shown in Figure 2 for each category respectively.

Of the patients in category 1 (“no risk regarding COPMI”), only 33% is caregiver. In other words, 2/3 of the patients who get the judgement “no risk”, are given this judgement *because they have no children*. Of the patients judged to be at risk because of their children (category 2), predictably almost all are caregivers for children (96%). Of the patients in category 3 (“COPMI check not carried out”), only an estimated 2% is caregiver. Thus, where professionals responded that they had not carried out the COPMI check, this was almost always *because their patients had no children*.

**Figure 3:**
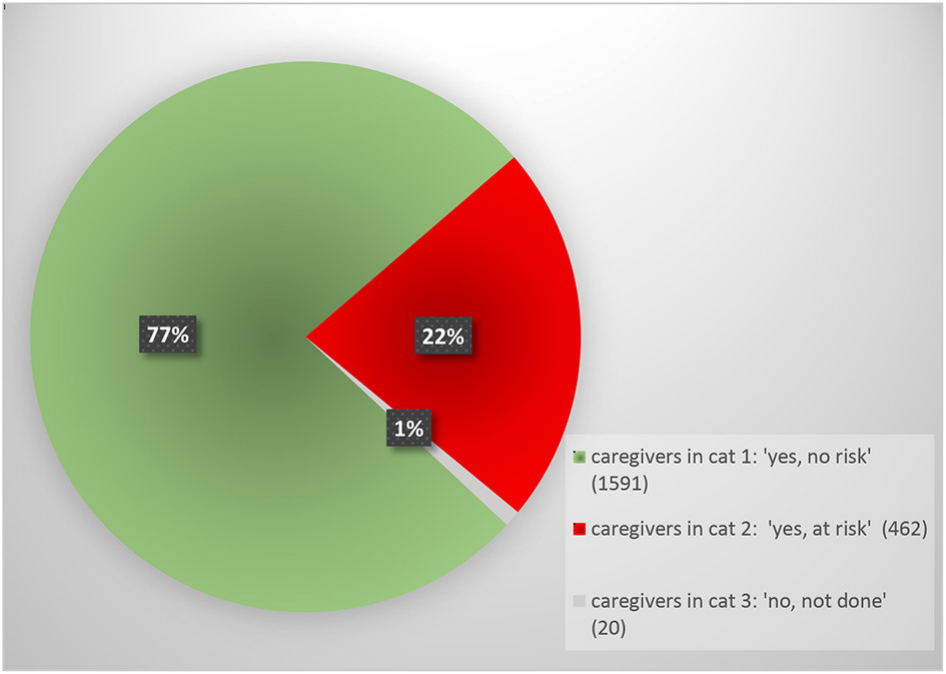
Answer to COPMI check question: caregivers only (*N* = 2,073).

Figure 3 shows how the COPMI check question was answered *for the caregivers only*. Because the patients without children have been filtered out, this presents more relevant numbers than Figure 1B.

### Content Analysis: Professionals' Assessment and Actions When Patients Are Caregivers

Two random samples (*N* = 75 each) were taken for patients in category 1 and 2, respectively. Below, we report on the files of the 97 patients in the samples that were caregivers (Table 1).

**Table 1:** Caregivers in the subsamples.

**Samples**	**Non-caregiver**	**Caregiver**
Subsample from category 1 (answer “no risk”) *N* = 75	50	25
Subsample from category 2 (answer “at risk”) *N* = 75	3	72
Total sample (*N* = 150)	53	97

For those patients who *were* caregivers of underage children, how did professionals estimate the risks to these children? Moreover, to what extent did they initiate further steps, including filing an official report to child abuse authorities and/or organizing help?

It was possible to categorize the situations that professionals encountered into five types of situations (Table 2). These situations were found both when there was judged to be risk or no risk.

**Table 2:** Types of situations encountered by mental healthcare professionals.

**Types of situations**	**Description**	**Number of patients with this type of situation**
1 Shared concern, some COPMI-related support already present	Professionals have concerns, patients share this concern to a degree. At the time of the intake, the children were already receiving treatment, or some parenting assistance organization was involved with the family. This includes situations where the patient is a divorced parent and his or her child lives with the other parent, while visitation takes place under the guidance of the Child protection agency.	43
2 Shared concern, no COPMI-related support present	Professionals have concerns, patients share this concern to a degree. At the time of the intake, there is no formal support given to family, children or parent regarding COPMI issues.	19
3 Shared concern, informal help seems present	There are concerns, but the patients rely on their social network to compensate.	4
4 Concern addressed by the professional, not shared by patients	Mental healthcare professional were concerned, however, patients emphasized that their vulnerabilities did not influence the well-being of their children. In some cases, children were mentioned as protective factor. In other cases contact with the children was very limited. Professionals seemed to agree with this.	9
5 Concern, without possibility to discuss with patients	Mental healthcare professionals were unable to discuss their concerns because patients avoid discussing the subject or discontinue treatment before any actions concerning the children could be taken.	7
6 Not enough information in files		15 (16%)

Within each type of situation, we found a variety in courses of action taken by professionals. Among these were the following:

- Regular discussion of the situation of the children with the patient
- Monitoring whether the situation seems to get worse
- Psycho-education about COPMI
- Referring the children to a COPMI training (not necessarily taken up)
- Coordinating with support organizations already involved with the family
- Organizing new help
- Addressing parental interaction as part of treatment
- Inviting the children to therapy sessions
- Inviting co-caregivers to therapy sessions
- Reporting to GP (either separately or included in the regular reports to the GP)
- Consulting with child abuse authority
- Reporting to child abuse authorities (Safe at Home).

Yet, among the 72 patients with children who were judged to be “at risk”, in 24 cases (33%) no actions were reported in the patient's file, even though we qualified even limited interventions as actions, as can be seen in the list above. For example in situation 1 (the situation “in which there are concerns, but some treatment or help is already in place”), there were no actions in 15 cases. Yet, in other cases in the same type of situation, professionals did undertake action. They would for example ask for permission to contact the other help involved, if this was not given they conferred with colleagues whether to break the confidentiality. If the permission was given, in some cases there was intensive coordination with the other support organizations, such as drawing up a safety plan together. Likewise, in the seven cases which we classified as situation 5 (“before concerns can be addressed, the patient discontinues treatment”), in three cases nothing was done after the patient stopped the treatment, while in the other cases the general practitioner was informed that there were concerns about the children. Overall, in each of the situations categorized above, supportive actions were initiated for about 2/3rd of the patients, yet in one out of three nothing was done.

### Focus Groups

The focus groups were used to help interpret and enrich the results from the file analysis. To find out more about the way professionals see their role and possibilities in relation to the COPMI and the COPMI check, focus groups started with a relatively open question:

“Given that you have an adult patient who is caregiver for (an) underage child(ren).

- What would you–as a mental healthcare professional–wish to be able to do in an ideal situation? And how could you be facilitated to do that?
- How do you experience the role of the Child check in this?”

1. The general line of answer was that professionals wished to have more information on what was really happening at their patients' homes. Several professionals mentioned that they wished they could talk to other people involved, including the partner, school, or neighbors, or doing home visits. In addition, the wish for some report from the GP was mentioned, so that they would know beforehand whether there were risks at home. A form of “truth finding” seemed to be the dominant focus for professionals when confronted with the COPMI check and the question of COPMI. Without extensive information on the situation of their patients and their children, most professionals refrained from taking any action, making this wish for “truth finding” an important barrier in arranging any help for COPMI.

2. Another theme which was often voiced was the concern of professionals to harm the therapeutic relationship, as they could feel intrusive and stigmatizing when asking about the children. This concern seems also related to the truth finding focus, as the fear is related to a continued probing into the situation of the patients' children. Some respondents felt that an obligatory set of questions might help them ask about their home situation without having to seem suspicious and thereby hurting the relationship. Some others felt the COPMI check already provided this role of legitimizing the probing questions.

3. The focus among some of the professionals on truth finding – as opposed to introducing support - became especially clear when the professionals were asked what they would do if they had enough information on their patients' children. One professional admitted not yet to have thought about that and another one answered “to follow the steps of the Reporting protocol”. Steering more toward the possibility of organizing help for the children of their patients, one professional said: “yes if you look at it that way, we should actually try to get every patient to have their children participate in a COPMI-prevention group”.

4. Some focus group participants addressed the possibility of initiating support more directly. They also mentioned some barriers. For example the fear that there would be long waiting lists in CAMHS was mentioned as barrier to even introducing the subject to their patients.

5. Reflecting on the COPMI check tool, some focus group members addressed it as positive, because it was part of step by step guide toward reporting in case of child abuse. None said it helped them choose a form of help to offer or initiate.

6. There were also remarks that shed light on the large group of patients for which the COPMI check was not done at all. Professionals mentioned that they sometimes forget it, or skip it for lack of time, Also, there is some irritation about the proliferation of obligatory instruments that professionals have to use, more seem to added all the time. The COPMI check (although maybe the most relevant of all, one person said) sometimes becomes submerged in the total of such obligations.

7. All in all, the issue of what to do when your patient has underage children seemed to provoke anxiety and a feeling of falling short with quite a few professionals.

8. All recognized the importance though, and none said that this should not be part of their work.

## Discussion

While many studies have studied the effectiveness of interventions for COPMI, this study is one of few focusing on improving access to such interventions, and focusing on the potentially very effective access through the parents (35). The Dutch “COPMI check” has enabled us to study in a focused way whether such a mandatory check contributes to the increase of support, prevention and (early) intervention for COPMI.

Our results showed that for a majority (58%, *n* = 8,337) of all Mondriaan patients aged 20–65, the COPMI check tool was not used at all by the professionals, a high percentage given that it is mandatory. Among these patients, we expect that there were many patients with underage children. We did not take a sample of this group to quantify this, but recent research showed that in Norway the number of outpatients with children was 36% (36, 37). A conservative estimate would therefore be that at least 25–30% of this group of patients. Thus, we conclude that for *at least 500–600 patients each year who do have children, the COPMI check question was not answered*. In other words, the COPMI check as operationalized at Mondriaan Mental Health Center, is still often either overseen or for some other reason not given attention. Previous research showed that mandatory instruments in mental healthcare can indeed be experienced as “a paper-filling exercise for managers” if insufficient argumentation and feedback is given concerning the added value of the instrument (38). Focus group discussions suggest that with the COPMI check as well, even though the importance of general issue of COPMI is recognized, using the tool is not always perceived as helpful, especially given the number of other obligatory instruments and rules that professionals nowadays are confronted with. In the total of such requirements, it can become unclear where the priorities of organizations lie.

Of course it is possible that for the (estimated) 2,000–2,500 patients with children for whom the tool was not used, the COPMI issue was still taken on, even though the tool was skipped. We have not been able to check this in the present study. Given the awareness and motivation regarding COPMI encountered in the focus groups, in combination with being overburdened by mandatory tools, it may well be that at times professionals did address the COPMI question even while neglecting the tool. Thus we *cannot* conclude that for all these patient the issues of their children was neglected. Yet, the numbers are high enough to give a worrying indication that too many COPMI problems may remain unseen.

For an additional 6% of all patients (*n* = 879), the mental health professionals explicitly responded that they did not do the COPMI check. This group of patients almost always did not have children, which was also the reason given for not carrying out the check. Thus by responding that they are “not doing the check”, in fact these professionals showed that they did check whether there were COPMI.

For the other 36% of patients aged 20–65, the COPMI check was carried out and patients were classified as either “risk” or “no risk” with regard to COPMI. In the no-risk group, we found that 66% the patients were not caregiver for underage children. Applying the COPMI check in those cases came down to stating that there was no risk, because there were no children involved. So in two-thirds of the cases where “no risk” was reported, the reason for this was the absence of any children. As we saw that other professionals whose patients had no children said that they had *not* done the COPMI check for that reason, we conclude that the wording and answering categories of the COPMI check question were multi-interpretable. This makes the quantitative data difficult to interpret without content analysis of the files to reveal parenting status. The multi-interpretability is also confusing to the user, as discussed and recognized in the focus group of the internal ambassadors. Recommendations will be necessary for a possible redesign of the tool, such as to reword the COPMI check, starting with a more basic question such as: “is the patient a caregiver for underage children or are there underage children in the household”. Among the *caregivers* for whom the COPMI check was carried out, the qualification “at risk” with regard to COPMI was given in only 22% of the cases. If “at risk” means that these children may develop lasting and serious emotional problems, then we know from research that the percentage is likely to be higher than 22%. A meta-analysis from 2012 (14) showed that one out of two (50%) COPMI develop a mental illness, with 32% developing a severe one. Possibly, professionals interpret “at risk” as meaning: immediate safety risk. We have already discussed how the COPMI check is introduced with a focus on safety and child abuse. The focus groups show that many professionals have taken on this narrow focus, which may lead to an underestimation of the *needs* of COPMI.

Analysis of the patient files of a random sample of patients was carried out. Focusing on caregiving patients for whom the COPMI check was carried out (*N* = 97), five types of situations encountered by professionals were identified, the most prevalent being “shared concern, some COPMI-related support already present”. We also identified a spectrum of actions that were taken by professionals to help support COPMI. However, for those COPMI judged to be at risk, in 33% of the cases no actions were taken at all. This is reason for concern, given the professionals' own judgement that there is risk (while, as mentioned above, that judgement itself already seems an underestimation). For the remaining 67% of patients on the other hand, there were examples of (sometimes relatively simple) sensitive and well informed actions to help COPMI. We conclude from this that in all the situations encountered by professionals, courses of actions are indeed available to them, as indeed the literature has shown as well (8, 39–41). Yet these actions toward support are still not readily or standardly carried out.

The focus group discussions shed light on the above. They showed that many professionals are more focused on truth finding (being clear whether there are severe problems at home) rather than on initiating support. We suggest that this focus comes from the fact that the COPMI check is presented as the first step in a protocol leading to the reporting of child abuse. As shown earlier, this protocol is concerned with “safety” rather than “needs” of COPMI. This study makes clear that this leads to a second bias, namely a focus on “deciding to report or not” (a focus that requires truth finding), vs. a focus on “organizing help”.

Contrasting a *safety/reporting focus* with a *needs/support focus* helps put into perspective the COPMI check and its limitations. Other studies have also warned for the consequences of limited focus on questions of safety and reporting (42, 43). From a historical and political perspective, the positioning of a COPMI check in terms of *safety/reporting* is understandable: it reflects the fact that public opinion, media and political urgency are often safety/reporting focused (43). But a COPMI check with such a focus at an AMHS institution limits the potential for COPMI. Rather than helping professionals to undertake basic supporting actions for COPMI in general, it sets professionals on a course to find out which children are at immediate safety risk and should be reported for child abuse. This requires truth finding, which is understandably a burden since an adult mental health professional is not in a likely position to undertake truth finding. With it comes a fear to harm the therapeutic relationship and appearing suspicious, as questions concerning children are more threatening when posed from the perspective of safety/reporting.

In contrast, CAMHS organizations, such as the Child and Youth division or the Prevention division at Mondriaan, are, by the nature of their daily work, more *needs/support focused*. That is, they are geared to helping a larger group of children that need support (among them COPMI), also where there is no direct threat to safety in a narrow sense. It has been noted by professionals in Mondriaan's CAMHS that very few referrals of children are prompted by AMHS professionals from Mondriaan–an observation that merits to be researched. Likewise, a training offered to COPMI at Mondriaan Mental Health Center still receives fewer participants than it can provide for. Of course one must at the same time be realistic about the fact that in practice, support is not always available to children even when they are adequately identified. Long waiting lists and limits to the funding are unfortunately still a limiting factor in CAMHS. Nonetheless, identification, basic help and referral are the first steps. Our study shows that these can be improved with a differently focused COPMI check. We would like to conclude with a few specific practical recommendations.

### Practical Recommendations

If the COPMI instrument could be redesigned toward a *needs/support focus*, a broader group of COPMI might be reached.

^*^Such a redesign would include basic guidance for professionals on how to initiate supportive actions, other than how to decide whether to report or not. We recommend that such practical guidance becomes part of the COPMI check.

^*^The guidance could make use of “best practice” examples, some of which we encountered in this study. Likewise the supportive actions that were taken up in 67% of the cases (as identified in this study), also provide a good starting point.

^*^CAMHS services should be given a role in the supportive actions, and in Mondriaan, where CAMHS and AMHS both take place within one organization, such collaboration could be institutionalized.

^*^Professionals should also be shown that they can take some supportive actions (for example: inviting the children to therapy sessions–possibly with help of colleagues from CAMHS) without having to know the exact extent of the problems at home. This would exempt them from some of the burden of truth finding. Possibly, such practical guidance on what to do next, once the professional has concluded that there are young children, would also contribute to a more widespread use of the COPMI check tool.

^*^The COPMI question(s) should be rephrased to resolve the multi-interpretability regarding patients that are not caregivers.

^*^AMHS organizations should carefully weigh the number of obligatory instruments that they introduce, in order for such instruments to retain their effectiveness.

### Strengths and Limitations

A strength of this research project was its setting in a large Mental Health Center that provides both CAHMS and AMHS and features a mandatory tool to check for the needs of COPMI. It has enabled us to include a large data set concerning 14,469 patients, evaluate the impact of a mandatory tool and see whether the presence of CAHMS in the same organization plays a role in helping COPMI. Furthermore, combining quantitative and qualitative data allowed us to avoid an unrealistic interpretation of the quantitative data, and brought to light biases in the instrument and the effects they had. The results lead to direct recommendations for the local situation.

A limitation of the study is that we evaluated a tool which is presented as part of a protocol aimed at reporting child abuse; this limits its effectiveness in stimulating help for COPMI. Therefore, other barriers to stimulating this help came less clearly into sight. Yet we feel that it is very important that we brought to light how confounding helping COPMI with reporting child abuse hampers the way COPMI can be helped through professionals working in AMHS.

We studied only one organization and the results are not representative for all Dutch organizations working with the COPMI check, nor of course for other countries working with other tools. Yet the organization studied is certainly relevant as it has implemented the COPMI check for a relatively long time and in a structured way, compared to other AMHS organizations.

Regarding the focus groups, it should be noted that only a limited number of professionals participated, moreover, the time spent on the issue was relatively short. These limitations did however enable us to talk to a relatively randomly selected and much larger group than if we had asked people to free a lot of time to talk about the COPMI check. The information we received was rich, varied, and useful. Another limitation of the focus group method is the risk of group-think evolving. We avoided this by our variant of the 1–2–4-All technique. Another limitation is that we cannot be sure to which degree the context (for example the presence of factual or perceived hierarchies within group participants) has influenced results: it may for example have led to participants paying more lip service to the importance of taking responsibility for COPMI than they are actually experiencing.

### Future Steps

It will be necessary to elaborate our study by focusing on those professionals who have focused more on helping than on reporting and have undertaken some action. One could then explore which barriers and enabling factors they encountered and by what means they could be assisted.

Our analysis of whether patients are caregivers for children touches upon the important question of parenting status among AMHS patients in general. Unfortunately we could not draw definite conclusions on this issue, as we did not have data on the parenting status of the large group of patients for whom the COPMI check question was ignored. A content analysis of a representative sample of this group would be a worthwhile future research endeavor.

Studying the files of patients where the COPMI check tool was *not* used would also give information about to which degree in these cases (serious) COPMI issues were missed.

Finally, it would be quite interesting to implement some of the recommendations that emerge from this research regarding the redesign of the COPMI check. This could then be evaluated as to whether more COPMI are receiving help, as well as with regard to whether professionals feel more supported in their task to take on COPMI issues.

## Data Availability Statement

The raw data supporting the conclusions of this article will be made available by the authors, without undue reservation.

## Ethics Statement

The studies involving human participants were reviewed and approved by Maastricht University Medical Ethics Committee. Written informed consent for participation was not required for this study in accordance with the national legislation and the institutional requirements.

## Author Contributions

SE, TA, and SL were involved in setting up the research and played an active role in the writing process. Data collection and analysis were done by SL and SE. All authors contributed to the article and approved the submitted version.

## Conflict of Interest

SE and TA are involved as employees at the mental healthcare facility where the research was conducted. The remaining author declares that the research was conducted in the absence of any commercial or financial relationships that could be construed as a potential conflict of interest.

## Publisher's Note

All claims expressed in this article are solely those of the authors and do not necessarily represent those of their affiliated organizations, or those of the publisher, the editors and the reviewers. Any product that may be evaluated in this article, or claim that may be made by its manufacturer, is not guaranteed or endorsed by the publisher.

## Footnotes

^1^ https://www.rijksoverheid.nl/onderwerpen/kindermishandeling/wat-kindermishandeling-is
